# Ten years of lesbian health survey research in the UK West Midlands

**DOI:** 10.1186/1471-2458-7-251

**Published:** 2007-09-19

**Authors:** Catherine Meads, Emily Buckley, Paul Sanderson

**Affiliations:** 1 Department of Public Health and Epidemiology, The University of Birmingham, Edgbaston, Birmingham, UK; 2 Centre for Health Psychology, Faculty of Health and Sciences, Staffordshire University, College Road, Stoke-on-Trent, ST4 2DE, UK; 3 Sexual Health Network Development, West Midlands South Strategic Health Authority, Osprey House, Albert Street, Redditch, B97 4DE, UK

## Abstract

**Background:**

Very little is known about the physical health needs of lesbian and bisexual women in the UK; most research has looked at mental or sexual health only. This article reports the results of four surveys carried out in the West Midlands between 1995 and 2005.

**Methods:**

The first two surveys were conducted in 1995–6 by a volunteer group, with participants from a lesbian health conference (n = 69) and in a convenience sample from a wide range of relevant groups and venues (n = 354). The second two surveys were commissioned by the West Midlands South Strategic Health Authority in partnership with the Gay Men's Health Network and were conducted in 2002 (n = 449) and 2005 (n = 166) and again used convenience sampling methods including the internet.

**Results:**

The mean age of respondents varied between 29–33 years and 5–7% were from a non-white ethnic background. The smoking rates varied from 42% o 55%, being twice the West Midlands regional average of 21% for women aged 16 or more. Similarly, problems with alcohol were reported in 25–37% of respondents, higher than the West Midlands regional average of 7% for women aged 16+. The prevalence of any mental health problem varied between 31–35% and any suicide attempt between 20–31%. Only 29–45% had revealed their sexual orientation to their GP and of these, approximately 50% had experienced a positive reaction.

**Conclusion:**

The results suggest health needs that current UK health services may not be meeting. There is a need to identify and target specific health measures for lesbians and bisexual women in order to ensure improved physical and mental health in the longer term.

## Background

Lesbian and bisexual women in the UK have a variety of physical, mental and sexual health needs that differ from those of heterosexual women, but there has been very little research that has been conducted in this area [1]. There are also no specific health services for lesbian and bisexual women; even though it has been acknowledged that gay and bisexual men benefit from distinct and specialised health services in the UK (such as gay men's health projects). Most research on lesbian, gay and bisexual health has been conducted in the USA, with smaller amounts in Australia, New Zealand and mainland Europe [2-4]. The research that has been conducted in the UK on lesbian and bisexual women has focused on mental and sexual health rather than physical health needs[5-8]. A recent review of the health needs of women who have sex with women discussed mainly sexual and mental health issues[9]. However, it also mentioned that the specific risk of cancers in this group has not been formally studied. Since this review was published, there has been a cohort study looking at a variety of cancers over 8 years' follow up in a sample of Danish men and women in registered homosexual partnerships[10]. They found that women in registered partnerships had similar cancer risks to the general female population except for a lower risk of cervical carcinoma in situ. The review also mentioned that the rates of smoking and consumption of alcohol were higher in women who had sex with women compared to heterosexual women but this was not referenced and no quantification of the higher rates were given [9]. The present paper aims to provide an indication of the rates of health behaviours and other indices of health in a population of lesbians and bisexual women in the UK.

Unfortunately there are considerable difficulties with conducting research within the lesbian and female bisexual community. There is no comprehensive list of all lesbians and bisexual women so no adequate sampling frame from which to take a random sample. Therefore, much survey research to date has relied on some form of non-probability sampling, also known as convenience sampling[6,8]. An exception to this is a consecutive sample used to survey participants attending UK GP practices[7]. However, that sample relied on participants being open about their sexual orientation and those who are open may differ from those who are not. The difficulty with convenience samples is the impossibility of knowing the generalisability to the population as a whole. On the other hand, in any health research, it is ethically important for potential participants to be allowed to refuse participation without having to give a reason. Random sampling techniques used when researching sensitive topics tend to have high levels of refusers and lesbians, gay men and bisexuals can refuse to participate in a very particular way – by not being open about their sexual preference. Therefore a random sample may not be as representative as one might at first assume[11]. One recent survey using probability sampling of British households found a prevalence of lesbians at 0.3% (and a drop-out rate of 28.5%)[12]. On the other hand, a recent population survey undertaken in the Netherlands found a prevalence of female homosexuality and bisexuality of 1.5% and 1.2% respectively. It is unclear whether a similar UK prevalence would have been found, using the same methodology[13] If the current UK government estimate that 5–7% of the population is homosexual (based upon a review of eleven population surveys conducted in USA, UK and the Netherlands) is true,[14] the UK survey found a very low prevalence[12].

This article reports the results of four surveys that were carried out in the West Midlands between 1995 and 2005. It investigates some aspects of physical and mental health within the lesbian and bisexual community and their use of health services.

## Methods

All participants were volunteers and not recruited via the NHS, either as staff or as patients so no ethical permission was required. However, ethical approval was sought and granted by Staffordshire University Ethics Committee for the fourth survey (Measure for Measure 2 conducted in 2005) as the University required that an ethical opinion be sought. Any person approached to participate in any of the surveys could decline by refusing to complete a questionnaire. All information was collected anonymously.

The first two surveys were conducted by a volunteer group called Lesbewell that was active in the Birmingham area between 1994–7 whose aim was to provide some health promotion for lesbian and bisexual women in the West Midlands. The group consisted of 12 volunteers of a variety of backgrounds including medicine, nursing and social care and organised a monthly newsletter and two lesbian health conferences in 1995 and 1996.

The first survey (Lesbewell 1) was conducted on participants at the second lesbian health conference, the aim being to discover participants' health needs and demographic information in order to help Lesbewell campaign for better health provision and information for lesbians. The second survey (Lesbewell 2) was part funded by Birmingham Health Authority and conducted between 1996 and 1997 on a much larger sample intended to be more representative of the lesbian population. There were four main aims of this survey: to identify health issues of interest to the lesbian community, to discover the preferred modes of health promotion on identified health issues, to discover how well the Lesbewell monthly newsletter was providing useful health information in terms of coverage and content and to investigate participants' experiences of GP and sexual health provision. The questionnaire was piloted first to establish whether the lesbian community would be amenable to a study of this type. The full questionnaire was distributed through the Lesbewell mailing list, through existing contacts with other groups (social groups, student groups, helplines, health groups, youth groups and sporting and activity groups), through personal contacts and private lesbian events and by visiting a wide range of gay and lesbian pubs and clubs. Every effort was made to involve as wide a cross-section of the lesbian community as possible.

The second two surveys (Measure for Measure 1 and 2, conducted in 2002 and 2005) were commissioned by the West Midlands South Strategic Health Authority in partnership with the Gay Men's Health Network. They were project managed by Paul Sanderson in association with University of Birmingham (Measure for Measure 1) and Staffordshire University (Measure for Measure 2). They were targeted at both gay and bisexual men and lesbians with the aim to establish the wider health needs of the lesbian, gay and bisexual population of the West Midlands. The questionnaires focused on demographics (gender, age, location and employment status), health indicators and factors known to affect health such as smoking, alcohol and substance abuse, experience of primary health care services, sexual health and mental health, plus an additional set of gender specific questions. Only the results for women respondents are presented here. Participants were obtained through convenience sampling; paper questionnaires were distributed through lesbian and gay service providers, social venues, mailing lists, support groups, lesbian and gay projects and contacts to help-lines. Measure for Measure 2 also had an online version of the questionnaire that was advertised on the internet via lesbian and gay websites and chat-rooms. Recruitment was restricted to participants aged over 16 years.

Examples of questions used in each of the surveys are shown in Table 1.

**Table 1:** Examples of questions used in questionnaires

	Lesbewell 1	Lesbewell 2	Measure for Measure 1	Measure for Measure 2
Sexual preference	Please indicate your sexuality. Lesbian Bisexual Other (please specify)	Would you describe yourself as.. Lesbian Bisexual Straight Don't know Other.....	How would you describe yourself? Lesbian, Gay, Bisexual, Heterosexual, Other	How would you describe your sexual orientation or sexuality – Lesbian, Gay, Bisexual, Heterosexual, Other
Smoking	I smoke		Do you smoke; how many per day; age started smoking:	Do you smoke; how many per day; ever tried to quit?
Problem with alcohol	I regularly drink heavily (more than 10 pints or equivalent a week or I would like to cut down/stop drinking)		Do you drink alcohol; how much in a typical week; do you think the amount you drink is a problem?	How often might you drink 6 or more units of alcohol in one session? 4 questions to assess 'problem' drinking'
Mental health problems/depression	I have experienced emotional/mental health problems		Have you ever been diagnosed with depression?	Have you ever been diagnosed with depression?
Suicide attempts			Have you ever thought of taking your own life; have you ever attempted to take your own life?	Have you ever thought of taking your own life; have you ever attempted to take your own life?
Eating disorders	I have/have had an eating disorder eg anorexia nervosa, bulimia nervosa		N/A	Have you ever been diagnosed with an eating disorder?
Out to GP	Are you out to your current GP? Yes/no	Are you out to him/her? Yes/no/don't know	Are you out to your GP?	Have you disclosed your sexuality to your GP?
GP reaction	If you have come out to your GP, please briefly describe the reaction you received. (Free text coded as positive, indifferent or negative.)	What response did you get when you came out? (Free text coded as positive, indifferent, negative or no explanation)	N/A	How did they react when they became aware?

## Results

A total of 423 questionnaires were analysed from the Lesbewell studies (69 from Study 1, and 354 from Study 2), and 615 from Measure for Measure (449 from Study 1 and 166 from Study 2).

The Lesbewell 2 survey originally had 378 questionnaires returned but 24 were excluded because they had no personal details (1), spoilt (5), were completed by the same person identically (11) or were from heterosexual women (7). The Measure for Measure 1 and 2 surveys included 22 and 32 heterosexuals respectively. These were retained in the analyses because all of the women who identified as heterosexual also had homosexual encounters, mostly in the previous 12 months. The higher proportion of women identifying as heterosexual in the last study may be due to the use of internet data collection, as this may have included women who were not open about their homosexual encounters and did not access the lesbian social scene.

Demographic information from the four surveys was remarkably similar (see Table 2). The mean age varied between 29.0 and 32.5 years and 4.9%–7.0% were from a non-white ethnic background. Between 66–71% of respondents were either employed or self-employed but the proportion who identified as health workers ranged from 5–40%. Lesbwell 2 included any health worker in this category whereas Measure for Measure 1 and 2 recorded NHS workers only. Inevitably the highest proportion of health workers was from the health conference but it is interesting to note that the majority attending the conference did not identify themselves as health workers. Most respondents lived in the West Midlands (72.4%–86.4%) and 15%–18% were parents.

**Table 2:** Demographic information

	Lesbewell 1	Lesbewell 2	Measure for Measure 1	Measure for Measure 2
Date	1996	1996–7	2002	2005
Number of respondents	69	354	449	166
Mean age (SD)	29.8 (8.3)	32.5$	29.0 (9.1)	31.7 (11.1)
Non-white ethnic background	5.8%	4.9%	6.2%	9%
Parent	15.9%	N/A	17.2%	15%
Live in West Midlands	72.4%	86.4%	N/A	91%
Employed	N/A	69.5%	66.2%	75%
Health worker	39.1%	28.0%	4.7%	13.3%
Identified as lesbian/gay	82.6%	81.6%	75.5%	67%
Identified as bisexual	14.5%	11.0%	18%	10%
Identified as heterosexual			5%	19%
Identified as 'other'			1.5%	4%
Mean age came out	21.3	N/A	N/A	19.85
Out before age 16	14.7%	N/A	N/A	24%

N/A – not available, $ – weighted mean calculated from categories.

With regard to sexual identity, the proportion who identified themselves as lesbian or gay women varied between 67–83% and the proportion of bisexual women remained relatively similar across the surveys at 10–18%. Lesbewell 1 and Measure for Measure 2 asked about when respondents had first identified themselves as lesbian or bisexual or at what age they started sexual relationships with women. The mean age that respondents came out (ie revealed their sexual orientation as lesbian or bisexual) was around 20 years old and between 15–24% came out before the age of 16.

### Health indicators

#### Health Risk Behaviours

Smoking rates found across the four surveys varied between 42%–55% and similar rates were seen in all surveys (see Table 3). The rates that respondents reported having a problem with alcohol varied between 25%–37%.

**Table 3:** Health indicators and screening results

	Lesbewell 1	Lesbewell 2	Measure for Measure 1	Measure for Measure 2
Smoke	42.0%	N/A	54.8%	48%
Problem with alcohol	26.1%	N/A	22.7%	37%
Obese	N/A	N/A	16.9%	15.7%
Eating disorder	15.9%	N/A	N/A	5%
Emotional/mental health problems or diagnosed depression	34.8%	N/A	31.4%	35%
Suicide attempts	N/A	N/A	31.3%	20%
Breast self examination	50.7%	N/A	53.9%	40%
Cervical smear attendance	55.1%	N/A	55.7%	60.8%

N/A – not available

#### Physical and mental health

The Measure for Measure 1 and 2 surveys recorded self-reported height and weight and body mass indexes were calculated (see Table 3). The rates of obesity (BMI 30+) were 16.9% (n = 402) and 15.7% (n = 139) respectively. Only Lesbewell 1 and Measure for Measure 2 collected information on self-reported eating disorder. The first survey found a high rate of 15.9% whereas the second found a lower rate of 5%. The prevalence of mental health problems was remarkably similar over the surveys at 31%–35% and the rate of suicide attempts varied between 20%–31%.

### Health screening

Two screening behaviours were recorded in both Lesbewell 1 and Measure for Measure 2 (see Table 3). The rates of breast self-examination were between 50.7% and 40% and for cervical screening attendance were 55.1% to 60.8%.

### General practice issues

Over 95% of respondents were registered with a GP. The proportion who revealed their sexual orientation as lesbian or bisexual to their GP varied between 29% to 45% (see Table 4). In Lesbewell 1, 46.4% stated that they had experienced homophobia from some part of the health service. In Lesbewell 2, the proportion of health workers who were 'out' to their GP was no different from the whole group. Just over 50% of participants in all surveys had a positive reaction from their GP only and this did not appear to change in the different surveys. However, the proportion with overtly negative reactions varied from 17.4% to 2%. From the Lesbewell 2 survey there were a variety of GP negative reaction quotes. Comments included 'would you like counselling?' 'there's plenty of time for a family yet' and 'its unnatural'. Other GPs responded by being 'aggressive and unhelpful', putting 'large red print on medical notes', by 'ignoring what I said and continuing to ask about contraception' and by putting 'note on doctor's file stating involved in perversion'.

**Table 4:** General practice results

	Lesbewell 1	Lesbewell 2	Measure for Measure 1	Measure for Measure 2
Registered with GP	94.2%	93.2%	N/A	96.2%
Out to GP	29.0%	35.9%	37.6%	45%
Positive GP reaction	52.2%	53.0%	N/A	51%
Indifferent GP reaction	30.4%	32.8%	N/A	46%
Negative GP reaction	17.4%	11.9%	N/A	2%

N/A – not available

## Discussion

Our findings are notably different from rates for similar problems found in routinely collected data. It is acknowledged that the methods of data collection are very different (interviews rather than questionnaire in some cases) so results may well not be comparable. However, the large differences for some outcomes are unlikely to be due to differences in data collection methods alone. For example, the smoking rates found in the surveys were twice the current West Midlands regional average of 21% (women aged 16 or over) [15] and the UK national smoking rate for single women aged 16 or over is 28%[16] (see Table 5). Also, having a problem with alcohol was more than double the current West Midlands regional average for women aged 16 or over of 7% (who drank more than six units on at least one day in the previous week) [15] and the equivalent UK national figure for single women aged 16 or over is 19% [16]. On the other hand, neither of Measure for Measure obesity rates were particularly higher than the West Midlands regional average of 15.6% (95%CI 15.2–16.1%) [17]. Also, the estimated UK prevalence rates for anorexia nervosa are 0.5–1% and for bulimia nervosa are 1–3% so there is little evidence for a higher rate of eating disorders in the Measure for Measure 2 survey [18]. It is possible that the high rate in the Lesbewell 1 survey was because it was undertaken at a health conference which may have preferentially attracted women with eating disorders.

**Table 5:** Comparison of results

Health indicator	Range from surveys	Previous UK research on lesbian and bisexual women	Similar results from routinely collected data
Smoking	42–55%	N/A	21% (W Midlands women aged 16+) 28% (UK single women aged 16+)
Alcohol	23–37%	N/A	7% (W Midlands women aged 16+) 19% (UK single women aged 16+)
Obesity	16–17%	N/A	16% (W Midlands women aged 16+) 19% (UK single women aged 16+)
Eating disorders	5–16%	N/A	0.5–1% Anorexia Nervosa, 1–3% Bulimia Nervosa (UK male and female)
Mental health problems/depression	31–35%	(RR 1.24)	10.8% mixed anxiety and depression (UK women)
Lifetime suicide rate	20–31%	N/A	14% (UK adults)
Breast self-examination	40–51%	40%	33–45% (UK female population)
Cervical smear attendance	55–61%	55%	82% (W Midlands women aged 25–64)

The prevalence of mental health problems found in the surveys (~35%) appears to be much higher than the 10.8% prevalence of mixed anxiety and depressive disorder for women in the general community [19] (see Table 5). Similarly, the lifetime attempted suicide rate (~20–30%) was higher than the national rate of 14% rate of adults who have considered suicide at some point in their lives [19].

It is estimated that the proportion of women in the general population that practice breast self examination is between 33% [20] and 45% [21] so the rates found here seem higher than average. However the 2002 estimate of the percentage of target population screened for cervical cancer for women aged 25–64 in the West Midlands is 82.0%, considerably higher than the proportion in both the Lesbewell 1 and Measure for Measure 2 surveys [22].

If the current UK government estimate of between 5–7% of the population being homosexual is correct,[14] in the West Midlands of approximately 2 million adult females this would equate to 120,000 lesbians and bisexual women. Therefore a survey of 449 women (Measure for Measure 1) may have surveyed less than 0.5% of the West Midlands population of lesbian and bisexual women.

There are considerable weaknesses that are inherent in cross-sectional surveys such as those presented here. For example, cross-sectional surveys by their nature cannot establish causation. Therefore, there can be no inference that being lesbian and bisexual is the causal reason why there were such high smoking rates because there is likely to be confounding factors such as social environment. The mental health problems category was self defined so could have been answered very differently by some respondents compared to others. The different surveys were carried out by different teams, using different methods which means that the results may not be comparable. The methodology used may not have been rigorously applied, particularly in the earlier surveys. Also the different definitions used to describe a variety of the measures can cause considerable uncertainty as to whether the results from one study are comparable with another. Another limitation was that definitions used in the surveys were not the same as those in the national statistics, even though every effort was made to find the most comparable definitions. Another weakness was the use of convenience samples and it is likely that the results have considerable selection and responder biases and so cannot give a very accurate picture of the health of lesbians and bisexual women in the West Midlands. For the first three surveys there were no records of the source of participant responses so it cannot be determined whether the samples obtained from, for example, sports and activity groups were any different from those obtained from helplines or support groups. Measure for Measure 2 recorded place of questionnaire completion and conducted sub-sample analyses to identify any differences. There were no differences between the different methods of paper questionnaire distribution, but there were differences between paper and online questionnaire completions. Women who completed the questionnaire online were less likely to be 'out' about their sexuality, less likely to smoke, and more likely to be obese and exercise less. Also, as all information was recorded anonymously, there is no way to tell whether the same participant completed more than one questionnaire.

The size of the four surveys varied considerably but many of the results were similar. The results possibly suggest a minority group with a less healthy lifestyle than the general population in the West Midlands, suggested by the considerably higher rates of smoking, alcohol and mental health problems. It is unclear as to the generalisability of these findings to the total population of lesbian and bisexual women in the West Midlands or in the rest of the UK. Unfortunately, there is very little published information with which to compare. Only a handful of surveys have been published, for example the UK Lesbians and Healthcare Survey 1997–8 [23]. This survey was conducted on 1066 lesbians living throughout the UK and only looked at breast self-examination, participation and experience of mammography and cervical screening and risk perceptions about breast and cervical cancer. The regular attendance for cervical screening was 55% and for breast self-examination was 40%, similar results to those found in the surveys presented here (see Table 5). There are no equivalent UK national surveys on the other aspects of physical and mental health and health behaviours discussed here and no currently published systematic reviews of UK lesbian health research findings.

There are potential implications of the smoking and alcohol results found. Since the self-report smoking rates are high, subsequent rates of cardiovascular diseases and cancer may possibly be correspondingly higher in lesbian and bisexual women. Similarly, the apparently high rates of alcohol intake may possibly cause an increase in corresponding diseases such as hepatitis and cirrhosis of the liver. It is currently unknown whether this is in fact the case, because there are no routinely measured details on sexual preference within hospital activity data or National Statistics survey data. As a result, if disease rates are higher within the lesbian and female bisexual population, this has remained largely hidden. There are currently plans to implement pilot projects examining the feasibility of sexual preference monitoring and this initiative is to be welcomed [24]. There is very little evidence to date as to the actual incidence of cardiovascular disease and cancers in lesbians and bisexual women in the UK. The best quality evidence to date is from a recently conducted cohort study based on the Danish homosexual partnership register linked to the Danish Cancer Registry [10]. There were 1,600 women included, which unfortunately was probably of insufficient power to detect relatively low incidence rates with statistically significance. There is also the problem that those women who are civil partnered may differ from those who are not (e.g. they may be more likely to be open about their sexuality). With the UK Civil Partnerships Act 2004 introduced, the same approach could be tried in the UK with a larger population. (There were already over 1000 female registered partnerships by 31^st ^January 2006 [25].)

However, one of the reasons why sexual preference has not been recorded in routinely collected information in the past is because it has been thought that many within the gay community will not be open about their identity for a variety of reasons including fear of discrimination. It is noticeable that in the 2005 Measure for Measure survey, 45% of respondents were out to their GP but the reasons for non-disclosure were not collected. It would be useful to collect reasons for non-disclosure to GPs or other parts of the health service in future research.

The apparent low rates of cervical smear attendance compared to the female population in the West Midlands may partly be in response to fear of a negative response. Qualitative research has suggested that approximately 38% of non-attenders do not attend because of negative aspects of the procedure [26]. Another possibility may be that some health care providers have suggested in the past that women who do not have sex with men are at low risk of cervical cancer so do not need to attend for cervical smears. This may be the reason for approximately 40% of non-attendance by lesbians [26] Future health education campaigns targeted specifically at lesbians could usefully address these perceptions and fears and medical professionals could be made more aware of the particular needs of their lesbian clients.

Regarding mental health, the findings of these surveys are similar to those in a recently completed study carried out in England and Wales that found a higher relative risk for psychological distress (RR 1.24, 95%CI 1.07–1.43) [6] in lesbian and bisexual women compared to heterosexual women. This is echoed in research from abroad finding higher suicide attempt rates in lesbians compared to heterosexual women (RR 2.06, 95%CI 1.71–2.48)[27]. There has been debate around the reasons for the apparently higher rates of mental health problems within the gay community. This has largely focused around whether problems found are largely intrinsically due to being homosexual or bisexual or whether because of societal factors such as discrimination. The change in the political climate in the UK provides us with an opportunity to test this out. The repeal of Section 28 of the Local Government Act, the implementation of the Employment Equality (sexual orientation) Regulations 2003, Civil Partnerships Act 2004. The Equality Act (Sexual Orientation) Regulations 2007 which outlaws  sexual orientation discrimination in the provision of goods and  services should provide an environment where overt discrimination gradually fades away. As a result, we may have a chance to find out whether the apparent current high levels of mental health problems and suicide attempts will gradually decrease.

A possible initiative to counter the apparently high rates of smoking and alcohol problems found in these surveys could be to run special anti-smoking groups and alcohol support group for lesbians and bisexual women only. There is anecdotal evidence that some lesbians and bisexual women are reluctant to attend support groups that are run principally for the majority heterosexual community, where they are likely the only one who is 'different' and that even where the health worker is 110% supportive and gay friendly, this feeling of difference may tend to put some lesbian and bisexual women off. In response to a high level of perceived demand, a lesbian sexual health service has been piloted in Glasgow[29]. Qualitative research would help to determine whether a similar smoking cessation service, for example, would fulfil a specific health need and better quality quantitative research may indicate whether there are sufficient numbers to warrant the provision of such a service.

Plainly, any overt discrimination within the health service is not acceptable and active steps have recently been taken to increase awareness of the issues [30-32]. It was gratifying that the homophobic comments that were published in the rapid responses section of the BMJ website [33] in response to the article about the health needs of women who have sex with women [9] were swiftly denounced by other health workers including the employer of one of the persons concerned. NHS professionals have a contractual duty to provide clinical care based on the best available evidence. Clearly there is a training issue for NHS employees which may have been partially addressed by the Knowledge and Skills Framework core dimension 6 covering equality and diversity. It is important that this is fully inclusive of lesbian and gay issues and related health indicator profiling. In order to fulfil this need it is important to have access to quality information on the health needs of the lesbian, gay and bisexual population. The survey research reported here aims to go some way towards filling the information gap that currently exists, but clearly much more and better quality research will be required in order to obtain a fuller picture.

## Abbreviations

BMI – Body Mass index

BMJ – British Medical Journal

GP – General Practitioner

NHS – National Health Service

RR – relative risk

UK – United Kingdom

USA – United States of America

## Competing interests

The author(s) declare that they have no competing interests.

## Authors' contributions

CM performed the Lesbewell surveys in 1995–7, completed the statistical analysis in 2006 and has drafted the manuscript and responded to peer review comments. EB performed the statistical analysis on the second Measure for Measure survey and advised and commented on the manuscript and peer review comments. PS conducted both of the Measure for Measure surveys and commented on the manuscript.

All authors have read and approved the final manuscript

## Funding

Lesbewell – Birmingham City Council, West Midlands South Strategic Health Authority. Measure for Measure – West Midlands south strategic Health Authority

## Pre-publication history

The pre-publication history for this paper can be accessed here:


